# Incidence of paediatric fatal and non-fatal low speed vehicle run over events in Queensland, Australia: eleven year analysis

**DOI:** 10.1186/1471-2458-14-245

**Published:** 2014-03-11

**Authors:** Bronwyn R Griffin, Kerrianne Watt, Belinda A Wallis, Linda E Shields, Roy M Kimble

**Affiliations:** 1 School of Medicine, The University of Queensland, St Lucia, Australia; 2 Queensland Children’s Medical Research Institute, Level 4, Royal Children’s Hospital, Herston Road, Herston, Queensland 4029, Australia; 3 School of Public Health, Tropical Medicine and Rehabilitation Sciences, James Cook University, Townsville, Queensland, Australia; 4 School of Population Health, University of Queensland, St Lucia, Australia; 5 Tropical Health Research Unit, James Cook University & Townsville Health Services District, Townsville, Australia; 6 Queensland University of Technology, Brisbane, Australia

**Keywords:** Child, Adolescent, Prevention & control, Accident prevention, Epidemiology, Queensland

## Abstract

**Background:**

The purpose of this study was to estimate the incidence of fatal and non-fatal Low Speed Vehicle Run Over (LSVRO) events among children aged 0–15 years in Queensland, Australia, at a population level.

**Methods:**

Fatal and non-fatal LSVRO events that occurred in children resident in Queensland over eleven calendar years (1999-2009) were identified using ICD codes, text description, word searches and medical notes clarification, obtained from five health related data bases across the continuum of care (pre-hospital to fatality). Data were manually linked. Population data provided by the Australian Bureau of Statistics were used to calculate crude incidence rates for fatal and non-fatal LSVRO events.

**Results:**

There were 1611 LSVROs between 1999–2009 (IR = 16.87/100,000/annum). Incidence of non-fatal events (IR = 16.60/100,000/annum) was 61.5 times higher than fatal events (IR = 0.27/100,000/annum). LSVRO events were more common in boys (IR = 20.97/100,000/annum) than girls (IR = 12.55/100,000/annum), and among younger children aged 0–4 years (IR = 21.45/100000/annum; 39% or all events) than older children (5–9 years: IR = 16.47/100,000/annum; 10–15 years IR = 13.59/100,000/annum). A total of 896 (56.8%) children were admitted to hospital for 24 hours of more following an LSVRO event (IR = 9.38/100,000/annum). Total LSVROs increased from 1999 (IR = 14.79/100,000) to 2009 (IR = 18.56/100,000), but not significantly. Over the 11 year period, there was a slight (non –significant) increase in fatalities (IR = 0.37-0.42/100,000/annum); a significant decrease in admissions (IR = 12.39–5.36/100,000/annum), and significant increase in non-admissions (IR = 2.02-12.77/100,000/annum). Trends over time differed by age, gender and severity.

**Conclusion:**

This is the most comprehensive, population-based epidemiological study on fatal and non-fatal LSVRO events to date. Results from this study indicate that LSVROs incur a substantial burden. Further research is required on the characteristics and risk factors associated with these events, in order to adequately inform injury prevention. Strategies are urgently required in order to prevent these events, especially among young children aged 0–4 years.

## Background

Preventable injuries to children are a significant burden on society and a considerable cost to the health care system [1]. “Low speed vehicle run over (LSVRO) incidents” is a term used to describe incidents where a pedestrian – usually a child – is injured or killed by a slow moving vehicle in either a traffic or non-traffic area [2]. LSVROs have been identified as a significant cause of transport pedestrian fatalities in young children. After pool drowning, LSVROs are the second largest cause of death from unintentional injury for children in Australia aged 1-4 yrs [3]. LSVRO incidents have different characteristics to other pedestrian casualties that occur on public roads and at greater speeds. However, comprehensive data on this particular mechanism have not been well documented. As most of these deaths and injuries are preventable and result when a parent or close relative is driving the vehicle, the cost in psychological terms for the family are high, often leading to extensive grief and frequently a breakdown of the family unit [4].

These types of incidents have been variously described in scientific journals since 1964, when a case report was described in Illinois [5,6]. Then later in 1980 a case series of patients admitted to intensive care at St Louis Children’s Hospital in Washington were reported [7]. International awareness grew during the 1990s with studies describing this tragic injury in the US, [8-14] Canada, [15] United Kingdom, [16] Brazil, [17] New Zealand [18-21] and Australia [22-25]. In Australia, child deaths in driveways were first highlighted as a significant health issue in 2000.

The World Health Organization recommends that data quality pertaining to transport injuries needs improvement in order to develop effective prevention strategies. Specifically, more comprehensive data about the numbers and types of injuries, and the circumstances in which those injuries occur, taking into account the complexities of the problem, are required. The information should be of sufficient quality to allow inferences to be made about the magnitude of problem, and where prevention measures are most urgently needed [26].

There is little current research that describes this mechanism of injury in detail, at a population level. No papers define both fatal and non-fatal data in the same data set, in the same area, in the same time period, with an age group up to 15 years [27]. In addition, different data collecting systems collect differing data (i.e., different fields of information), so ascertaining the true incidence of these events is difficult.

Currently, the most frequent method of identifying LSVRO incidents is to identify non-traffic pedestrian transport incidents using ICD codes, then manually interrogate data to determine the location of the incident (if it is recorded). This level of difficulty in classifying LSVRO incidents has compromised ability to complete comprehensive, population-based studies, as the process is complex, time consuming and costly.

These methodological limitations of previously published work on LSVRO events have resulted in a lack of adequate information about the burden (fatal and non-fatal) of this injury, and the circumstances surrounding the incident. This information is crucial in informing effective prevention strategies to reduce the burden of LSVRO events.

The purpose of this study was to provide improved estimates of the incidence of fatal and non-fatal LSVRO events at a population level. Characteristics of LSVRO events are addressed elsewhere [28].

## Methods

This study was a population-based, retrospective cohort study. For the purposes of this study, the definition of LSVRO suggested by the Commission for Children Young People and Child Guardian (CCYPCG) and Queensland Injury Surveillance Unit (QISU) has been used. That is, LSVRO incidents are those where a pedestrian, usually a child, is injured or killed by a slow moving vehicle (30 km/hr or 19 mph) in both traffic and non-traffic areas (2). All children aged 0–15 years (inclusive) involved in a fatal or non-fatal LSVRO incident between 1999–2009 were included in this study.

### Ethics approval

This study was approved by the Behavioural and Social Sciences Ethics Review Committee of the University of Queensland; Children’s Health Service District – Health Research Ethics Committee, Office of Health and Medical Research – Queensland Health. Approval to access data was also obtained from all of the relevant data custodians (Queensland Ambulance Service; Queensland Health; Mater Hospital; Commission for Children, Young People and Child Guardian).

In order to calculate incidence of LSVRO events as accurately as possible, retrospective data on all fatal and non-fatal LSVROs among 0–15 year olds in Queensland, Australia from 1999–2009 were obtained from multiple sources across the continuum of care and manually linked by the first author. Any child who received treatment for an LSVRO from one of the following were included in the study:

1) Pre-hospital (Queensland Ambulance Service-QAS) - statewide.

2) Emergency Department (Emergency Department Information System - EDIS) – almost statewide, supplemented by Queensland Injury Surveillance Unit - QISU);

3) Hospital admissions for 24 hours or more (Queensland Health Admitted Patient Data Collection) - statewide; and

4) Fatalities (Commission for Children, Young People and Child Guardian -Child Death Review (CCYPCG-CDR) – statewide. The CCYPCG-CDR did not exist prior to 2004, so for 1999–2001 data were obtained from the Queensland Council on Obstetric and Paediatric Morbidity and Mortality (QCOPMM).

QAS, CCYPCG and QHAPDC data are state-wide. Every incident that resulted in death, admission to a hospital in Queensland, or that was attended by Queensland Ambulance Service, is included in this data set. EDIS is not statewide but does capture the majority of ED presentations in QLD, and all of the major Emergency Departments.

Identification of relevant cases was difficult due to the absence of specific codes describing LSVRO incidents. Data extraction occurred separately for each of the databases. Data were first obtained on children admitted to hospital as a consequence of an LSVRO, using ICD codes (external cause, and where possible, location codes – see Table 1). In cases where it was not possible to determine whether the incident was an LSVRO from the available ICD codes, further information was sought from manually searching additional databases (e.g., QISU, EDIS) using text fields to rule out high speed incidents. Data regarding all definite incidents resulting in hospital admission were then obtained from QAS, EDIS and QISU, if the incident was present in that database, using manual linkage. In addition, all cases involving paediatric pedestrian crashes were extracted from the pre-hospital database and manually interrogated using free text fields (case description). Similarly, all paediatric pedestrian events in the EDIS database were extracted using the presenting complaint field, and this field was then further manually interrogated for relevant cases. For QISU data, a similar manual search process occurred for all cases initially extracted using transport codes (0509–0599). For fatality data, the events were already clearly defined in the CDR database, using a combination of coroners’ reports and forensic investigation forms. For all databases, where insufficient information existed to determine whether a case was definitely an LSVRO incident, it was not included. Once all definite LSVRO incidents were identified in each database, data were linked using (in order) name, gender, age/date of birth, date of incident, and hospital. Data were then cleaned to ensure that every incident was represented only once in the database. A more detailed explanation regarding case ascertainment and data linkage is described elsewhere [29].

**Table 1:** **ICD codes used for retrospective data search across all time periods **[30-33]

**Code set of ICD version**	**External cause code**	**Place of occurrence code**
ICD9-CM (1/1/1999)	E813.6, E814.7, E816.6, E816.7, E817.7, E819.6, E825.7	E849.0, E849.5, E849.8
ICD-10-AM 1st Ed (1/7/99 – 30/6/00)	V03 (all),V04 (all), V09, V13, V14, V19, V84.1, V84.2, V84.3, V84.4, V84.6, V84.7, V84.9	N/A
ICD-10-AM 2nd Edition - 6th Edition (30/6/00 - current)	V03, V04, V09, V13, V14, V19, V84.1, V84.2, V84.3, V84.4, V84.6, V84.7, V84.9	Y92.0, Y92.4, Y92.7, Y92.8
ICD-10-AM 3rd edition - 6th Edition (1/7/02-current)	V03.0, V03.1, V03.9, V04, V09.0, V09.1, V84, V84.1, V84.2, V84.3, V84.4, V84.5 V84.8	Y92.00, Y92.40, Y92.41, Y92.42, Y92.48, Y92.49, Y92.87

### Data analysis

Crude incidence rates (IRs) and 95% Confidence Intervals [34] were calculated separately for fatal and non-fatal LSVRO incidents (this was further divided into events resulting in hospital admission, and events not resulting in hospital admission). The Australian Bureau of Statistics (ABS) provides population data for each age year, from 1901 onwards. Number of LSVRO events for a particular age and calendar year were divided by the corresponding population for that age year, for each year from 1999–2009. This was done separately for fatal and non-fatal events (hospital admissions and non-admissions), all events combined, males and females, and for the total population. Incidence rates were also calculated for age groups (0-4 years; 5-9 years; 10-15 years). Data on all LSVRO events that occurred in Queensland were extracted, however only events that involved residents of Queensland were used to calculate incidence rates, because accurate population data (for the denominator) on non-Queensland residents was not available. Trends over time were analysed by chi-square test for trend using Epi Info (7.0).

## Results

**Figure 1:**
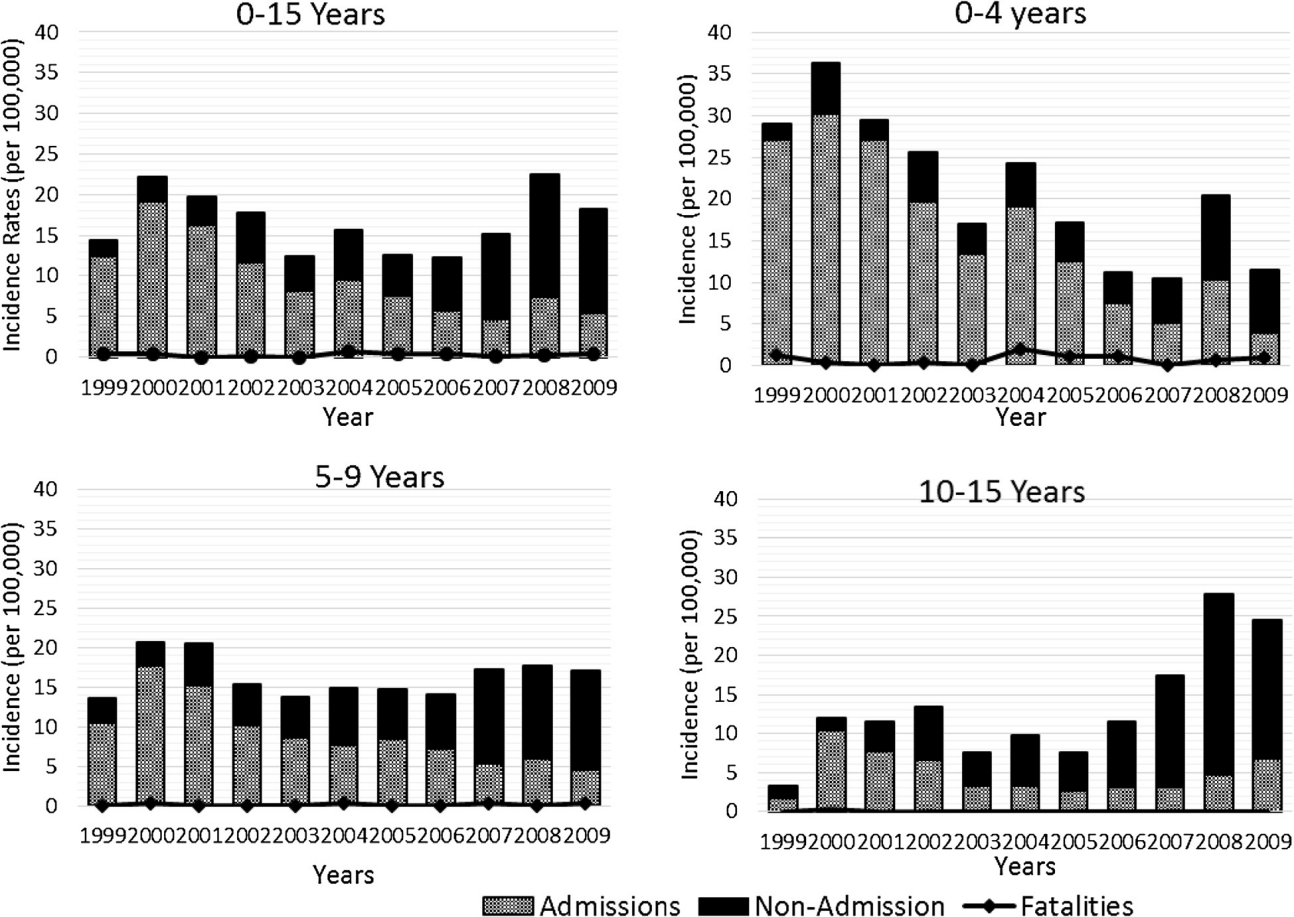
Crude LSVRO fatal, admission and non-admission incidence rates by year, age-group, 1999–2009, Queensland (per 100,000 per annum).

Incidence rates for fatal, non-fatal and total LSVRO events by calendar year (1999–2009) are shown in Figure 1. During the 11 year data collection period, there were 1,611 LSVRO incidents among 0–15 year old residents of Queensland, yielding an annual crude incidence rate of 16.87 per 100,000 (male: 20.97/100,000; female: 12.55/100,000). Approximately 39% (n = 621) of the total number of LSVRO events involved 0–4 year olds, yielding an annual incidence rate of 21.45/100000/annum, and incidence was higher among males (IR = 26.75/100,000; females: 15.85/100,000). The annual incidence rate for 5–9 year olds was 16.47/100,000 (males 20.72; females 11.99), and for 10–15 year olds it was 13.59/100,000 (males 16.62; females 10.40).

Over the 11 years of data collection, the annual incidence rate for non-fatal LSVRO events (16.60/100,000) was 61.5 times higher than for fatal events (0.27/100,000). Of all non-fatal events, 56.8% (n = 896) involved admission to hospital for 24 hours or longer (IR = 9.38 per 100,000 per annum). As can be seen in Table 2, incidence rates were higher among boys than girls for all age groups, for both fatal and non-fatal events. Table 2 also shows that the incidence rate was higher in 0–4 year olds than other age groups, for both fatal and non-fatal LSVRO events. Between 1999–2009, 85% of fatal and 38% of non-fatal events involved children aged 0–4 years. Similar patterns were observed in admissions and non-admissions (this includes treatment involving pre-hospital or Emergency Department only).

**Table 2:** Crude incidence rates of fatal and non-fatal LSVRO events by age-group and gender (1999–2009) (per 100,000, per annum)

**Age groups**	**Fatal**			Non-fatal*		
	**Male IR**	**Female IR**	**Total IR**	**Male IR**	**Female IR**	**Total IR**
	**(95% CI)**	**(95% CI)**	**(95% CI)**	**(95% CI)**	**(95% CI)**	**(95% CI)**
0-4 yrs	0.94	0.49	0.72	26.55	15.84	20.969
	(0.940-0.941)	(0.496-0.498)	(0.725-0.726)	(26.509-26.593)	(15.824-15.875)	(20.946-20.993)
5-9 yrs	0.19	-	0.10	20.98	12.05	16.63
	(2.034-2.048)			(20.951-21.016)	(12.039-12.076)	(16.619-16.656)
10-15 yrs	0.05	-	-	16.66	10.45	13.59
	(0.052-0.053)			(16.646-16.692)	(10.444-10.473)	(13.577-13.603)
0-15 yrs	0.43	0.15	0.293	21.01	12.59	16.77
	(0.428-0.429)	(0.1506-0.151)	(0.292-0.294)	(20.997-21.033)	(12.579-12.601)	(16.769-16.789)

*Non-fatal IR includes admission to hospital for 24 hours or longer, as well as treatment at Ambulance and/or, Emergency Department only).

### Trends over time

Between 1999–2009, the incidence of LSVRO events (fatal and non-fatal combined) among 0–15 year olds increased from 14.79 (1999) to 18.56 (2009) per 100,000, with two distinct peaks (Figure 1). The first peak occurred in 2000 (22.48 per 100,000) and the second in 2008 (22.65 per 100,000). The lowest observed IR of LSVRO events occurred in 2003 (12.36 per 100,000) and 2006 (12.54 per 100,000). The increase in LSVRO incidence over time was not significant (X^2^ = 0.05; p > .05). Of concern is that in the four years since 2006, the incidence of LSVRO events increased by a factor of almost 1.5, to 18.56 per 100,000. Trends over time differed by severity of injury: incidence of hospitalisation resulting from LSVRO events decreased over the 11 year period (X^2^ = 96.80; p < .001); but incidence of non-admissions increased (X^2^ = 172.97; p < .001). Incidence of fatalities did not change significantly (X^2^ = 0.15; p > .05).

In 0–15 year old males, the incidence of fatal and non-fatal LSVRO events (combined) increased from 18.39 to 23.82 per 100,000 between 1999–2009 (an increase by a factor of 1.5, although this increase was not significant (X^2^ = 0.93; p > .05). For females, the incidence increased from 10.98 to 13.00 per 100,000 (X^2^ = 0.91; p > .05). For most years between 1999–2009, the incidence of LSVRO events among 0–15 year old females in Queensland remained between 9 and 14 per 100,000 per annum. For both males (X^2^ = 96.73; p < .001) and females (X^2^ = 41.81; p < .001), incidence of hospital admission due to LSVRO incidents decreased during the 11 year period, but incidence of non-admissions increased (males: X^2^ = 60.97; p < .001; females: X^2^ = 64.27; p < .001). Mortality did not change significantly during this time.

As indicated in Figure 1, over the 11 year period, annual incidence of LSVRO events among 0–4 year olds decreased significantly from 30.3 to 12.51 per 100,000 (incidence among males decreased from 35.09 – 14.68/100,000/annum, and among females incidence decreased from 25.24 – 10.2/100,000/annum). Conversely, annual incidence of LSVROs among children aged 5–9 years increased from 13.62 in 1999 to 17.8 in 2009 (females: IR increased from 7.99 to 13/ 100,000/annum; males: IR increased from 18.39 to 22.45/100,000/annum), however these increases were not significant. A more substantial increase was observed among older children aged 10-15 yrs (X^2^ = 77.58; p < .001). In 1999 the annual incidence rate was 3.29 per 100,000 (males: 4.49 per 100,000; females: 2.03 per 100,000), and in 2009 it was 25.13 per 100,000 (males: 32.7; females: 16.07). Among this age group, most of this increase appears to have occurred since 2005.

Trends over time differed by severity, gender and age group. As can be seen in Figure 1, incidence of admissions due to LSVRO events among 0–4 year old boys (X^2^ = 75.45; p < .001) and girls (X^2^ = 57.81; p < .001) decreased significantly between 1999–2009, as well as for 5–9 year old boys and girls (X^2^ = 26.92, p < .001; X^2^ = 4.90; p < .01, respectively). Incidence of admissions resulting from LSVRO events increased significantly among 10–15 year old boys (X^2^ = 3.94; p < .05), and although an increase was also observed among girls, this trend was not significant (X^2^ = 0.29; p > .05) in each age group (p < .001) (Table 3). The incidence of fatal events among female children aged 0-4 years increased from 0.84 to 1.36 per 100,000, per annum, but decreased among boys of this age group (1.59 to 0.68 per 100,000 per annum); neither trend was significant. It is important to keep this in context. There were seven deaths from LSVRO events among 0–15 year old females in Queensland between 1999–2009, and all of these deaths occurred in the 0–4 year age group. The remaining 19 deaths from LSVRO events occurred among male children, and 14 of these were in boys aged 0-4 years.

**Table 3:** Crude incidence rates and 95% CI by age groups, gender and calendar year

**0-4 years**						
**Incidence of FATAL LSVRO events**						
	**Male**		**Female**		**Total**	
	**IR**	**95% CI**	**IR**	**95% CI**	**IR**	**95% CI**
1999	1.60	1.590-1.600	0.84	0.839-0.843	1.23	1.226-1.230
2000	0.80	0.793-0.798	0	0	0.41	0.406-0.410
2001	0	0	0	0	0	0
2002	0	0	0.82	0.821-0.825	0.40	0.398-0.402
2003	0	0	0	0	0	0
2004	2.28	2.274-2.292	1.60	1.597-1.608	1.95	1.946-1.957
2005	2.23	2.222-2.240	0	0	1.15	1.144-1.147
2006	1.45	1.446-1.454	0.77	0.763-0.768	1.12	1.116-1.119
2007	0	0	0	0	0	0
2008	1.34	1.340-1.347	0	0	0.69	0.689-0.693
2009	0.64	0.636-0.641	1.36	1.355-1.363	0.99	0.987-0.988
**Incidence of nonfatal LSVRO events resulting in hospital admission**						
1999	32.71	32.527-32.884	23.55	23.423-23.685	28.25	28.142-28.362
2000	39.00	38.792-39.217	23.50	23.367-23.628	30.23	30.112-30.348
2001	38.53	38.319-38.737	17.43	17.338-17.529	27.06	26.951-27.160
2002	22.59	22.473-22.714	16.47	16.382-16.562	19.62	19.543-19.693
2003	17.72	17.628-17.816	9.78	9.726-9.830	13.47	13.414--13.515
2004	23.60	23.472-23.722	14.43	14.351-14.505	19.13	19.059-19.203
2005	14.13	14.058-14.204	11.77	11.710-11.834	12.60	12.555-12.648
2006	10.88	10.822-10.932	3.83	3.812-3.848	7.45	7.424-7.477
2007	9.14	9.091-9.180	2.24	2.227-2.245	5.06	5.047-5.081
2008	12.10	12.039-12.157	9.26	9.214-9.306	10.37	10.338-10.410
2009	7.02	6.991-7.056	0.68	0.677-0.682	3.95	3.938-3.963
**Incidence of nonfatal LSVRO events not resulting in hospital admission**						
1999	1.60	1.590-1.600	3.36	3.348-3.381	2.46	2.449-2.464
2000	7.96	7.919-8.00	3.36	3.340-3.372	5.72	5.698-5.739
2001	3.15	3.130-3.159	1.66	1.654-1.666	2.42	2.415-2.430
2002	10.13	10.075-10.181	2.47	2.460-2.481	6.41	6.382-6.429
2003	5.39	5.367-5.420	2.44	2.434-2.455	3.96	3.947-3.973
2004	3.81	3.788-3.823	6.41	6.379-6.445	5.08	5.058-5.093
2005	5.95	5.921-5.979	3.14	3.125-3.153	4.58	4.566-4.597
2006	5.80	5.773-5.829	2.30	2.288-2.307	4.10	4.084-4.111
2007	8.43	8.391-8.474	2.98	2.968-2.994	5.79	5.768-5.807
2008	11.43	11.371-11.482	8.55	8.506-8.590	10.03	9.994-10.063
2009	7.02	6.991-7.056	8.16	8.117-8.195	7.57	7.547-7.597
**5-9 years**						
**Incidence of FATAL LSVRO events**						
	**Male**		**Female**		**Total**	
	**IR**	**95% CI**	**IR**	**95% CI**	**IR**	**95% CI**
1999	0	0	0	0	0	0
2000	0.74	0.743-0.748	0	0	0.38	0.381-0.385
2001	0	0	0	0	0	0
2002	0	0	0	0	0	0
2003	0	0	0	0	0	0
2004	0.72	0.721-0.726	0	0	0.37	0.368-0.372
2005	0	0	0	0	0	0
2006	0	0	0	0	0	0
2007	0.70	0.694-0.699	0	0	0.36	0.355-0.359
2008	0	0	0	0	0	0
2009	2.04	2.033-2.048	0	0	1.05	1.046-1.048
**Incidence of nonfatal LSVRO events resulting in hospital admission**						
1999	15.16	15.080-15.238	6.39	6.361-6.426	10.89	10.852-10.932
2000	28.35	28.198-28.496	7.10	7.061-7.134	18.02	17.950-18.085
2001	20.68	20.571-20.786	9.38	9.328-9.425	15.19	15.131-15.243
2002	12.44	12.379-12.506	7.75	7.712-7.791	10.16	10.127-10.201
2003	10.92	10.860-10.970	6.13	6.103-6.164	8.59	8.556-8.617
2004	12.31	12.248-12.372	3.80	3.782-3.817	8.16	8.129-8.186
2005	10.76	10.708-10.815	6.04	6.005-6.064	8.46	8.427-8.487
2006	12.02	11.963-12.083	2.23	2.219-2.236	7.25	7.220-7.270
2007	7.67	7.632-7.706	4.40	4.3824.423	6.08	6.057-6.098
2008	8.96	8.918-9.005	5.82	5.791-5.847	7.43	7.407-7.458
2009	5.44	5.418-5.468	4.30	4.281-4.321	4.89	4.871-4.903
**Incidence of nonfatal LSVRO events resulting in hospital admission**						
1999	4.55	4.526-4.569	1.60	1.593-1.603	3.11	3.102-3.122
2000	3.73	3.712-3.747	2.37	2.356-2.375	3.06	3.057-3.076
2001	5.17	5.144-5.194	5.47	5.442-5.497	5.31	5.297-5.333
2002	3.66	3.643-3.676	6.98	6.941-7.011	5.27	5.252-5.288
2003	5.09	5.069-5.117	6.90	6.865-6.934	5.97	5.952-5.994
2004	6.52	6.485-6.548	6.84	6.805-6.874	6.67	6.651-6.697
2005	7.89	7.853-7.930	4.53	4.504-4.547	6.25	6.229-6.272
2006	6.37	6.334-6.395	7.43	7.389-7.463	6.88	6.859-6.906
2007	13.25	13.182-13.313	9.54	9.492-9.588	11.44	11.401-11.482
2008	13.10	13.033-13.162	9.46	9.410-9.540	11.32	11.286-11.366
2009	17.01	16.926-17.094	7.89	7.846-7.924	12.56	12.522-12.611
**10-15 years**						
**Incidence of FATAL LSVRO events**						
	**Male**		**Female**		**Total**	
	**IR**	**95% CI**	**IR**	**IR**	**IR**	**95% CI**
1999	0	0	0	0	0	0
2000	0.63	0.629-0.633	0	0	0.32	0.322-0.326
2001	0	0	0	0	0	0
2002	0	0	0	0	0	0
2003	0	0	0	0	0	0
2004	0	0	0	0	0	0
2005	0	0	0	0	0	0
2006	0	0	0	0	0	0
2007	0	0	0	0	0	0
2008	0	0	0	0	0	0
2009	0	0	0	0	0	0
**Incidence of nonfatal LSVRO events resulting in hospital admission**						
1999	2.56	2.555-2.574	0.68	0.675-0.680	1.65	1.643-1.651
2000	15.79	15.712-15.863	4.67	4.648-4.691	10.38	10.346-10.416
2001	10.54	10.487-10.586	4.59	4.567-4.608	7.65	7.620-7.670
2002	8.52	8.481-8.559	5.11	5.088-5.134	6.55	6.524-6.566
2003	5.34	5.314-5.360	1.25	1.245-1.251	3.34	3.334-3.354
2004	3.47	3.457-3.485	3.05	3.036-3.061	3.27	3.256-3.275
2005	2.26	2.253-2.269	2.98	2.970-2.994	2.61	2.605-2.619
2006	4.47	4.452-4.489	1.77	1.760-1.771	3.15	3.144-3.162
2007	3.32	3.309-3.335	2.91	2.901-2.923	3.12	3.113-3.131
2008	6.58	6.550-6.606	2.89	2.875-2.898	4.78	4.766-4.795
2009	8.18	8.141-8.211	5.73	5.701-5.750	6.70	6.681-6.722
**Incidence of nonfatal LSVRO events resulting in hospital admission**						
1999	1.92	1.917-1.930	1.35	1.351-1.358	1.65	1.643-1.651
2000	1.89	1.888-1.901	1.33	1.330-1.338	1.62	1.618-1.626
2001	4.96	4.936-4.980	2.62	2.611-2.632	3.82	3.811-3.834
2002	9.13	9.087-9.171	4.47	4.452-4.492	6.86	6.835-6.879
2003	4.74	4.723-4.764	3.74	3.727-3.759	4.26	4.243-4.269
2004	7.52	7.488-7.554	5.49	5.463-5.512	6.53	6.510-6.552
2005	5.65	5.630-5.678	4.18	4.158-4.193	4.93	4.919-4.949
2006	7.27	7.234-7.297	9.42	9.375-9.460	8.31	8.287-8.339
2007	13.84	13.779-13.903	14.56	14.494-14.627	14.19	14.146-14.237
2008	27.41	27.287-27.535	18.48	18.391-18.560	23.06	22.985-23.133
2009	25.07	24.961-25.187	10.88	10.829-10.927	18.15	18.092-18.208
**0-15 yrs**						
**Incidence of FATAL LSVRO events**						
	**Male**		**Female**		**Total**	
	**IR**	**95% CI**	**IR**	**IR**	**IR**	**95% CI**
1999	0.48	0.482-0486	0.26	0.253-0.257	0.37	0.371-0.374
2000	0.72	0.716-0.719	0	0	0.37	0.367-0.370
2001	0	0	0	0	0	0
2002	0	0	0.25	0.244-0.247	0.12	0.118-0.120
2003	0	0	0	0	0	0
2004	0.90	0.903-0.905	0.48	0.474-0.477	0.70	0.6945-0.697
2005	0.67	0.664-0.667	0	0	0.34	0.340-0.343
2006	0.44	0.435-0.438	0.23	0.228-0.231	0.34	0.334-0.337
2007	0.21	0.213-0.216	0	0	0.11	0.109-0.111
2008	0.42	0.418-0.421	0	0	0.22	0.214-0.217
2009	0.41	0.409-0.412	0.43	0.432-0.435	0.42	0.420-0.423
**Incidence of nonfatal LSVRO events resulting in hospital admission**						
1999	15.73	15.682-15.776	9.45	9.420-9.476	12.67	12.646-12.700
2000	26.79	26.712-26.872	11.11	11.081-11.148	18.80	18.758-18.838
2001	22.17	22.108-22.239	9.97	9.945-10.004	15.88	15.847-15.913
2002	13.98	13.936-14.017	9.34	9.310-9.365	11.60	11.575-11.624
2003	10.78	10.753-10.814	5.32	5.306-5.336	8.01	7.991-8.023
2004	12.21	12.174-12.243	6.66	6.642-6.680	9.51	9.486-9.525
2005	8.43	8.408-8.455	6.55	6.529-6.566	7.40	7.386-7.415
2006	8.73	8.705-8.753	2.53	2.522-2.534	5.71	5.698-5.720
2007	6.43	6.416-6.450	3.17	3.158-3.174	4.62	4.614-4.632
2008	9.03	9.004-9.053	5.76	5.749-5.780	7.33	7.319-7.347
2009	6.98	6.962-6.999	3.69	3.676-3.695	5.27	5.263-5.282
**Incidence of nonfatal LSVRO events resulting in hospital admission**						
1999	2.66	2.655-2.668	2.04	2.038-2.047	2.36	2.356-2.365
2000	4.31	4.294-4.317	2.27	2.268-2.279	3.32	3.311-3.323
2001	4.48	4.470-4.494	3.24	3.233-3.2502	3.88	3.871-3.886
2002	7.69	7.665-7.709	4.67	4.656-4.682	6.22	6.206-6.231
2003	5.05	5.034-5.061	4.35	4.342-4.365	4.71	4.701-4.719
2004	6.10	6.087-6.121	6.19	6.168-6.203	6.14	6.132-6.156
2005	6.43	6.417-6.452	3.98	3.965-3.986	5.24	5.227-5.247
2006	6.55	6.5296.565	6.67	6.647-6.684	6.60	6.592-6.617
2007	12.01	11.975-12.042	9.50	9.473-9.527	10.79	10.766-10.809
2008	18.06	18.008-18.108	12.64	12.602-12.673	15.42	15.390-15.452
2009	16.84	16.790-16.883	9.11	9.080-9.130	13.08	13.050-13.101

## Discussion

The results of this study indicate that the incidence of LSVRO events is higher among younger children aged 0–4 years, and among males. Of the three children injured in an LSVRO every week in Queensland, two are male. The majority of the total burden from LSVRO events related to non-fatalities, yet this is the area least previously researched.

This is the first study to date in which population-based data across the continuum of care (pre-hospital to fatality) have been linked to obtain an accurate estimation of the magnitude of fatal and non-fatal paediatric LSVRO events. While several studies exist in which LSVRO events are described, [18,20,21,24,35] none have been as extensive as this population-based, eleven year retrospective review.

Incidence rates for LSVRO events have been reported in five previous studies; three of these studies were conducted in Auckland, New Zealand, [19,21,35] and the other two in the USA (Washington State [15]; and Utah [36]). Three studies (all from New Zealand) reported non-fatal incidence rates among 0–15 year olds, [19,21,35] in all three studies incidence rates were between 7–9 per 100,000 per annum. The non-fatal incidence rates estimated in the present study are higher than that previously described in the literature [15,19,21,35]. One other study, conducted in Utah, estimated that the non-fatal LSVRO incidence rate in children aged less than 10 years was 7.09 per 100,000 per annum. However after the authors conducted analyses to counteract the acknowledged methodological limitations, the re-calculated incidence rate was closer to that estimated in the present study. It is likely that the higher observed incidence in the present study is due to more accurate identification of total number of LSVRO events than has occurred in previous studies, and inclusion of non-fatal LSVRO events from pre-hospital and emergency department data. We considered it important to include such events because injuries sustained from LSVRO events that do not result in hospitalisation may still be serious, and are often treated on an out-patient basis.

Four of the five previously published studies reported fatal incidence rates [15,19,21,35]. Three of these studies (all from Auckland, New Zealand) involved children aged 0-15 years. In each of these studies, mortality rates were higher than the annual incidence rate of 0.27 per 100 000 estimated in the current study (IR:0.63 per 100 000, [21] IR:0.64 per 100,000, [35] IR: 0.77 per 100,000 [19]). In the fourth study, incidence of fatal LSVRO events among 1–4 year olds in Washington State from 1979–1983 was estimated at 3.2 per 100 000. It is unlikely that fatal LSVRO cases were missed in the present study. Fatality data were obtained from the Child Death Review database maintained by the Commission for Children, Young People and the Child Guardian, which records extensive details of all deaths among children in Queensland, from coroners’ reports, police reports, and other sources. One explanation could be that over time the management of the trauma patient has improved globally.

The study conducted in Washington State was the only study in which gender-specific fatality incidence rates were reported (male 1–4 year olds: 3.2 per 100,000; female 1–4 year olds: 2.5 per 100 000), and again these incidence rates were higher than that observed in the present study. No other study has reported gender and age specific incidence rates for LSVRO events. However, males have consistently been described in descriptive studies as being more frequently involved in LSVRO events in which gender has been analysed [18,21,24,37]. This is consistent with our findings. No other studies in the literature have reported age-specific non-fatal LSVRO incidence rates. However, in descriptive studies that have involved comparable age groups to the present study, higher frequencies of non-fatal LSVRO events have been reported among children aged 0–4 years [18,24]. The results of the present study confirm this.

In the present study, incidence of total LSVRO events among children aged 0–15 years increased over the 11 year period between 1999–2009. It is not possible to compare this with previous research as no other study to date has presented data on trends over time. Between 1999–2009, two “dips” are apparent, the first of these occurred in 2003. It is possible that this reduction in LSVRO events could be in relation to a driveway safety campaign that was released at that time in the state of New South Wales [38]. The second dip in 2005–2006 may be the result of a non-fatal (but serious/critical) incident that occurred in Australia in October 2005 involving the child of a national sporting celebrity. This event, and the long recovery period, was highly covered by the media at the time [39].

The present data also indicate that while incidence of LSVRO events decreased over time for children aged 0–4 years, an increase was observed among older children aged 10–15 years. The reasons behind this require further investigation.

This study has inherent strengths in comparison to other previously published studies on incidence of LSVRO events. Firstly, this study was a population-based study. Three of the five previously published studies occurred in Auckland, New Zealand, which is predominantly an urban population. The present study included all possible cases across the state of Queensland (population = 4,560,100 (19.7% 0–15 year olds), 1.73 million km^2^). It is also possible that car movement behaviour in urban areas may be different to that in a rural setting [40]. Secondly, this study captured all possible incidents across the continuum of care, from pre-hospital to fatalities. Thirdly, the case definition for LSVRO events in the present study is broader than that used in previous studies. LSVRO events do not have an assigned ICD code. These events are not restricted to a certain location. Hence authors are forced to search for their own definitions. To date, the two most popular codes used to search for LSVRO events employed in previous studies are: 1. place of occurrence codes (e.g. driveway), or 2. external cause codes (e.g. non-traffic pedestrian). In studies where driveway location is used to identify LSVRO events, [36,41] case counts exclude events that occur in any location other than the driveway (e.g., in traffic areas where a vehicle may be travelling slowly, such as a school drop off zone, or other non-traffic areas where a vehicle may travel slowly such as beaches, parks, car parks, recreation areas, etc.). Hence, incidence rates from these studies are not directly comparable to our study. Studies in which LSVRO events have been identified through non-traffic pedestrian codes are limited because speed is not defined within ICD codes and LSVRO incidents can occur in traffic areas. Ours is the first study to not limit location (driveway) or external cause (non-traffic pedestrian).

Finally, time is a possible explanation for the observed differences in previously published work on incidence of LSVROs, and our study. Two of the five other studies were conducted in the 1980s, and are therefore not directly comparable to the results of the current study.

There were some limitations associated with this study. Analyses were limited by availability of data – this is especially the case for non-fatal LSVRO incidents. While data on fatalities, hospital admissions and pre-hospital treatment that resulted as a consequence of LSVROs were complete, data on children who sought treatment at an emergency department is less complete. EDIS and QISU do not encompass all emergency departments in Queensland. Hence, our estimation of non-fatal LSVRO events may be an under-estimate. Secondly, apart from QISU (an injury surveillance database), the databases accessed for this study are administrative databases. Therefore important data regarding circumstances leading to the event are not routinely or consistently recorded.

## Conclusions

This is the most comprehensive, population-based epidemiological study on fatal and non-fatal LSVRO events to date. This study employed sophisticated methodology to ensure the most accurate ascertainment of LSVRO events possible, and resulted in estimation of age- and gender-specific incidence rates over an 11 year period. Results from this study indicate that LSVROs incur a substantial burden. The initial call for change is to unify consistent definitions between at least local databases, and to implement an ICD code that refers uniquely to this event. Further research is required to fully comprehend the risk factors associated with LSVRO incidents, and to inform appropriate intervention strategies. Specifically, information on culture, geographical location of incident, dwelling design, vehicle design, supervision prior to the event, family composition, etc., is required. This could be captured through mandatory reporting of these event types, however, this would need to be accompanied by some mechanism to ensure that sufficient data were collected consistently across the continuum of care. Optimally, a purpose-built, prospective data collection system would facilitate capture of this information. Utilizing additional injury and trauma databases to supplement data on admitted patients and children fatally injured would also further assist with the ascertainment of LSVRO incidents. Strategies are urgently required in order to prevent these events, especially among young children aged 0–4 years.

## Abbreviations

ABS: Australian Bureau of Statistics; CCYPCG: Commission for Children, Young People, & Child Guardian; CDR: Child Death Review; ED: Emergency Department; EDIS: Emergency Department Information System; ICD: International Classification of Diseases; IR: Incidence Rate; LSVRO: Low Speed Vehicle Run Over; QAS: Queensland Ambulance Service; QCOPMM: Queensland Council on Obstetric and Paediatric Morbidity and Mortality; QHAPDC: Queensland Health Admitted Patients Data Collection; QISU: Queensland Injury Surveillance Unit.

## Competing interests

The authors have no competing interest to disclose.

## Authors’ contributions

BRG: Ms Griffin conceptualized and designed the study, carried out the analysis, drafted the initial manuscript and approved the final manuscript as submitted. KW: Associate Professor Watt carried out quality control of analysis, reviewed and revised the manuscript and approved the final manuscript submitted. BAW: Ms Wallis assisted in the design of the study and approved the final manuscript as submitted. LES: Professor Shields reviewed and revised the manuscript and approved the final manuscript. RMK: assisted in conceptualization of the study, reviewed and revised the manuscript and approved the final manuscript as submitted. All authors read and approved the final manuscript.

## Authors’ information

Bronwyn Griffin: BNursing (PGDip ACNE), and PhD student with University of Queensland.

Kerrianne Watt: A/Professor Epidemiology and Public Health, James Cook University; PhD BSc (Hons).

Belinda A Wallis: BBuilt Env; Injury Prevention Research Officer with Centre for Children’s Burns and Trauma Research, Brisbane, Queensland, Australia.

Linda E Shields: MD, PhD, FACN, Professor of Nursing – Tropical Health, James Cook University, Townsville, Australia.

Roy M Kimble: MD, MBChB, FRCS, FRACS, Director of Paediatric Burns and Trauma Surgery, Royal Children’s and Mater Children’s Hospitals, Brisbane, Australia.

## Pre-publication history

The pre-publication history for this paper can be accessed here:

http://www.biomedcentral.com/1471-2458/14/245/prepub
